# Long-term succession in a coal seam microbiome during *in situ* biostimulation of coalbed-methane generation

**DOI:** 10.1038/s41396-018-0296-5

**Published:** 2018-10-15

**Authors:** Sabrina Beckmann, Alison W. S. Luk, Maria-Luisa Gutierrez-Zamora, Nur Hazlin Hazrin Chong, Torsten Thomas, Matthew Lee, Michael Manefield

**Affiliations:** 1 School of Chemical Engineering, University of New South Wales, High Street, 2052, Sydney, NSW Australia; 2 Charles Perkins Centre, School of Life and Environmental Sciences, University of Sydney, Camperdown, Sydney, NSW 2006 Australia; 3 Centre for Marine Bio-Innovation, School of Biological, Earth and Environmental Sciences, University of New South Wales, High Street, 2052, Sydney, NSW Australia; 4 School of Biosciences and Biotechnology, University Kebangsaan Malaysia, 43600, Bangi, Selangor Malaysia; 5 School of Civil and Environmental Engineering, University of New South Wales, High Street, 2052, Sydney, NSW Australia

**Keywords:** Microbiology, Environmental microbiology

## Abstract

Despite the significance of biogenic methane generation in coal beds, there has never been a systematic long-term evaluation of the ecological response to biostimulation for enhanced methanogenesis *in situ*. Biostimulation tests in a gas-free coal seam were analysed over 1.5 years encompassing methane production, cell abundance, planktonic and surface associated community composition and chemical parameters of the coal formation water. Evidence is presented that sulfate reducing bacteria are energy limited whilst methanogenic archaea are nutrient limited. Methane production was highest in a nutrient amended well after an oxic preincubation phase to enhance coal biofragmentation (calcium peroxide amendment). Compound-specific isotope analyses indicated the predominance of acetoclastic methanogenesis. Acetoclastic methanogenic archaea of the *Methanosaeta* and *Methanosarcina* genera increased with methane concentration. Acetate was the main precursor for methanogenesis, however more acetate was consumed than methane produced in an acetate amended well. DNA stable isotope probing showed incorporation of ^13^C-labelled acetate into methanogenic archaea, *Geobacter* species and sulfate reducing bacteria. Community characterisation of coal surfaces confirmed that methanogenic archaea make up a substantial proportion of coal associated biofilm communities. Ultimately, methane production from a gas-free subbituminous coal seam was stimulated despite high concentrations of sulfate and sulfate-reducing bacteria in the coal formation water. These findings provide a new conceptual framework for understanding the coal reservoir biosphere.

## Introduction

Gas produced by biological processes (biogas) has a large role to play in meeting rising energy needs worldwide [1]. Coal-bed methane (CBM) is a relatively untapped energy source representing up to 20% of the world’s biogas resources [2–4] and is contained in actively mined and abandoned coal reservoirs globally [5]. The increased utilization of this unconventional natural gas source can significantly reduce the environmental drawbacks of coal-fired power plants [6, 7] prompting commercial enterprises to focus on the enhancement of biogenic methane production. To date, only incremental improvements of *in situ* biogas yields from coal have been observed [5, 8].

Biogenic methane is the product of ongoing microbial coal degradation whereas thermogenic methane is formed during the coalification process [9]. Mixed biogenic (δ^13^C value less than −60‰) and thermogenic (δ^13^C value greater than −50‰) signatures [10, 11] have been observed in many coal reservoirs worldwide [5]. The yield of biogenic methane is dependent on the degree of coal biodegradation and the availability of metabolic products generated from bacterial activity such as methanol, H_2_ and acetate used by methanogenic archaea to produce methane [12–19].

Biogenic methanogenesis in coal reservoirs is slow *in situ* and needs to be stimulated to be economically viable [12]. Nitrogen (N) and phosphorus (P) are essential nutrients for microbial metabolism and are limiting in many methanogenic environments [20, 21], thus reducing the effectiveness of biogas production. The applications of multi-nutrient amendments (N, P, vitamins, trace elements) or methanogenic substrates (acetate and/or CO_2_/H_2_) has been trialled in field-scale pilot tests but are not cost-effective for commercial implementation, and the use of compounds that can be used by methanogens can result in the enhancement of coal-independent methanogenesis [22].

One successful strategy in the bioremediation of groundwater contaminants is the application of passive oxygen-releasing material, e.g. calcium peroxide that releases oxygen upon contact with water (2CaO_2_ (s) + 2H_2_O - > 2Ca(OH)_2_ + O_2_ (g) [23]. The oxidation of coal or aerobic hydrocarbon degradation processes can drive the release of soluble organics into the coal formation water which can then be further biodegraded by microorganisms into methanogenic substrates [13, 17]. Despite the practical significance of *in situ* stimulation of the microbial communities in coal reservoirs, there has been no systematic long-term study of how nutrient and oxygen treatments alter microbial communities over the production life time of a coal gas well. Earlier *in situ* amendments were applied on productive methane-generating coal sites or non-productive lignite reservoirs under low or depleted sulfate concentrations ( < 1 mM; [24, 25]). Waters rich in sulfate (2–5 mM) occur in many coalbed aquifers [24] but are generally not found in association with methane given sulfate reducing bacteria (SRB) can outcompete methanogenic archaea for substrates such as acetate [26].

Additional work is required to answer several important practical and fundamental questions which are significant in the context of treatments for enhanced methane production of coal reservoir microbiomes. Can an indigenous microbial community in sulfate-rich coal formation water be stimulated by the addition of basic nutrients ammonium (N) and phosphate (P)? Can an initial oxic incubation phase in addition to nutrient amendment reshape the coal-reservoir microbial community in favour of methane generation? Are there systematic changes in microbial community composition and ecological function driven by treatment strategies? What are the metabolic drivers of methane generation? The answers to these questions have important implications for the enhancement of methane generation from productive and non-productive coal reservoirs for gas enterprises worldwide. They also offer insights into fundamental aspects of the coal reservoir microbiology such as the effects of high sulfate concentration on the methane production regime, the ability of methanogenic communities to overcome intermittent oxygen intrusion and the effects of basic nutrient limitation on the coal associated subsurface microbiome.

**Fig. 1:**
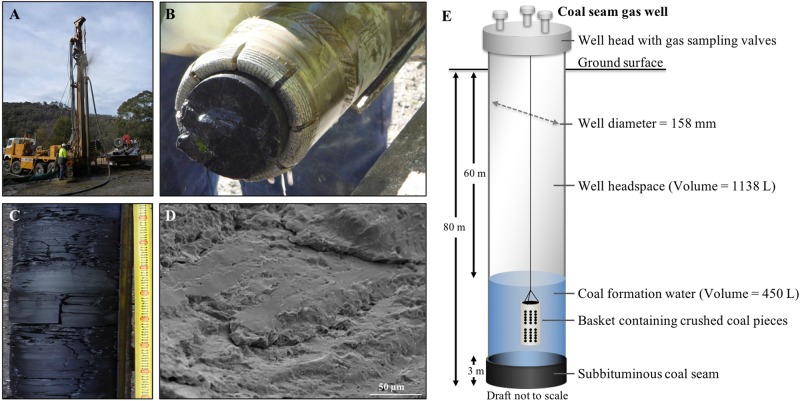
Subbituminous coal-seam, located in the western coalfields of NSW, field conditions for a long-term *in situ* methane stimulation trial. **a** Drilling of gas wells. **b**, **c**Coal cores after drilling. **d** Scanning electron microscopy of coal surface. **e** Schematic of the coal gas well

A field trial to stimulate methane production was carried out in a gas-free sulfate-rich subbituminous coal-seam reservoir located in the western coalfields of New South Wales, Australia. Our aim was to assess the impact of basic nutrient addition and an oxic/anoxic treatment regime on the biogeochemistry and microbiology of the coal formation water. A system free of biogas enabled us to clearly ascribe biogenic methane production to treatment effects through exclusion of treatment effects on release of pre-existing methane. Four coal gas wells extending 80 m below ground into a 3 m thick subbituminous coal seam (Fig. 1a, E) were used to assess three treatment strategies. In the first treatment, the nutrients ammonium chloride (N) and potassium phosphate (P) were applied to approximately 450 L formation water in contact with the coal seam. In the second treatment, in addition to nutrients calcium peroxide was provided during the first three months to initiate oxic conditions in the formation water, and subsequently removed to initiate an anoxic phase. The third treatment was a positive control amended with the methanogenic substrate sodium acetate (20 mM) and nutrients. A fourth well was left untreated as a negative control for comparison.

## Materials and methods

### Gas well setting and amendments

To stimulate the in situ biological conversion of coal to methane gas, four wells (bore holes) were drilled to 80 m depth, reaching 3 m into a subbituminous coal seam of the Sydney Basin at the Lithgow State Coal Mine, NSW, Australia (Fig. 1). All wells were lined with an iron casing and secured by backfilling with concrete. Wells were developed over 12 months by removing groundwater on a monthly basis and allowing them to refill (approx. 500 L influx per 24 h) to ensure the concrete mineralogy had no impact on the microbiology. The well diameter was 158 mm and the total well volume was 1588 L (1138 L well headspace and 450 L coal formation water – equilibrated with the aquifer in which the seam is submerged). The well head contained 3 gastight apertures for gas sampling. The field trial was conducted for 15–18 months starting in July 2011. The formation water submerged the coal and the lower 20 m of each well. The water temperature was 16 °C with a pH range of 7.8–8.7. Prior to the field trial, sodium bromide (Unilab Ajax, Australia) was added to the well water and monitored for decreases over time. Decreases were commensurate with the removal of water volume during sampling. This confirmed that the coal seam is poorly permeable (no hydraulic conductivity or water flow through the coal seam) and that there was no connectivity between the wells. To assess the impact of nutrient addition and aerobic/anaerobic treatment on the microbial community, water chemistry and methane production, the following treatments were applied: Well 1, nutrients + acetate (positive control); well 2, nutrients and calcium peroxide for oxygen release in the initial 3 months, well 3, nutrients only, and well 4, no amendment (negative control). Nutrients included 1.8 mM NH_4_Cl (Univar Ajax, Australia) and 1.9 mM Na_2_HPO_4_, (Unilab Ajax, Australia). Acetate (20 mM; Sigma Aldrich, Australia) was added to assess if the in situ conditions were generally suitable for methane production. The oxygenic treatment involved the addition of 100 mg calcium peroxide (Sigma Aldrich, Australia) contained in a fine mesh inside a permeable PVC canister, connected to the bottom of a larger permeable PVC canister containing coal pieces of the size of ~3 cm^3^ per coal piece taken from the original coal cores and placed in the formation water such that coal pieces could be retrieved for microbial community characterisation and the peroxide treatment could be terminated by removal of the connected canister (Fig. 1). The water sampling was accomplished using a bladder pump (PVC 3/8in discharge, ThermoFisher Scientific, Australia) deployed with a stainless steel drop tube (ThermoFisher, Australia) and operated at a low flow refill ratio of 40:20 to avoid the removal of dissolved gases in the formation water. The drop tube was lowered such that the bladder pump drew groundwater samples from the aqueous volume within the coal seam in each well. The pump and the connecting tubes were drained and flushed between wells to avoid the carriage of water between well sampling. Formation water used for chemical (anions, cations) and microbiological (cell counts) analyses were sampled monthly over a period of 15–18 months. Samples were immediately processed in the laboratory as outlined below.

### Coal and water chemical analyses

The coal composition was analysed at the Bureau Veritas Australia Pty Lt, Cardiff, NSW, Australia. Scanning electron microscopy (SEM) was performed according to Hazrin-Chong, Manefield [27]. Coal formation water was filtered through a 0.2 μm syringe filter (Millipore, Australia) and aliquots for cation analyses were subsequently acidified with formic acid (10% v/v; Sigma-Aldrich, Australia) to a pH < 2 and stored at −20 °C until further processing. Anion (F, Cl, Br, NO_2_, NO_3_, PO_4_, SO_4_) and cation (B, Ca, Fe, K, Mg, Mn, Na, P, S, Si) concentrations were analysed using ion chromatography (IC) with conductivity detection and inductive coupled plasma optical emission spectroscopy (ICP-OES) at the Mark Wainwright Centre (UNSW, Sydney, Australia). The instrument detection limits were 0.02 mg/L for B, Si and Mn, 0.2 mg/L for Fe and P, 0.5 mg/L for Ca, K, Mg, Na and S, 2 mg/L for F, NO_2_, NO_3_ and Br, and 5 mg/L for Cl and PO_4_.

### Oxidation-reduction potential (ORP), pH and temperature analyses

ORP, pH and temperature of the formation water were monitored monthly over a period of 15–18 months using an EcoSense pH100A meter connected to a pH/Temperature and ORP (Ag/AgCl) probe (YSI, Xylem, Australia). The formation water was pumped out of the well using a bladder pump (Grundfos Pumps Pty. Ltd, Australia). The water was collected in a sampling cell and probes were submerged into the water. An average of 10 readings were taken after every refill of the pump and until temperature equilibration.

### Total methane, acetate and sulfate analyses

Total methane (headspace and dissolved), acetate and sulfate concentrations were monitored monthly. Gas samples were taken from the well head apertures and were transferred directly into 10 mL gastight serum vials using a gastight glass syringe until further processed for CH_4_ and stable isotopes analyses (Intertek Geotech, Australia) as per Whiticar [28]. Dissolved methane was analysed according to Kampbell and Vandegrift [29]. Methane was analysed using a Shimadzu GC-2010 gas chromatograph with flame ionization detection (GC-FID) fitted with a GASPRO PLOT column (60 × 0.32 mm; Agilent Technologies, Australia). The carrier gas was helium (3 mL min^−1^), inlet temperature was 250 °C. The oven temperature program was isothermal 100 °C (1 min) and then 25 °C min^−1^ to 250 °C and held for 1 min. Gas samples of 100 μL were withdrawn directly from the sampling flasks using a pressure lockable gastight glass syringe (SGE Analytical Science, Australia) and injected into the GC. To describe the gas distribution throughout the well headspace columns samples were taken from the top of the well and 10 m and 30 m below ground level. Methane concentrations displayed a linear trend with increasing concentration. This linear relationship was used throughout the trial to calculate methane concentrations in the columns using the methane concentration in samples taken from the top of the wells. For the analyses of acetate and sulfate concentrations, water was filtered through a 0.2 μm syringe filter (Millipore, Australia) and subsequently 900 µL filtered water was acidified with 100 µL 10% v/v formic acid (Sigma-Aldrich, Australia) to a pH < 2. Acetate (1 µL) was analysed by GC-FID (Shimadzu, Australia) using a DB-FFAP column (30 m x 0.32 mm; Agilent Technologies, Australia). Carrier gas, helium (1 mL min^−1^). Injection port 250 °C, split mode (1:30). Oven temperature: 60 °C for 1 min and then 15 °C min^−1^ to 250 °C. Sulfate was measured by ion chromatography with conductivity detection (Mark Wainwright Centre, UNSW, Australia). Carbon dioxide was not monitored as the seam rests in a mineral carbonate subsurface matrix.

### Cell counts

Coal formation water samples were immediately fixed by the addition of glutaric dialdehyde (0.2 μm filtered, 2% final concentration; Sigma Aldrich, Australia) and stored at 4 °C in the dark. Prior to analysis, an aliquot was diluted 1000 fold in particle-free phosphate-buffered saline (0.9 g of NaCl, sodium phosphate buffer 15 mM, pH 7.4, 0.2 μm filtered), thoroughly shaken and transferred to a microscopic slide that was treated with a mounting medium (9.6% polyvinylalcohol 4–88 moviol (Sigma-Aldrich, Australia) in 24% glycerol (Sigma-Aldrich, Australia) prior to applications. Cells were stained using SybrGreen I (Invitrogen, Australia), and counting was performed using a BX51 epifluorescence microscopy (Olympus, Australia) according to Lunau et al. [30].

### DNA extraction

Coal formation water (3–20 L) was filtered through a 0.1 μm Supor®polyethersolfone (PES) membrane disc filter (Pall Corporation, Port Washington, NY) that was subsequently cut into quarters and stored in a mixture of 5 mL RNA later® Stabilization Solution (Life Technologies, Carlsbad, CA, USA) and 5 mL 1 × TE buffer (10 mM Tris-HCL, 0.1 mM EDTA; pH 8) at −20 °C until further processing. DNA was extracted from one quarter of the filter using phenol–chloroform extraction as described by Lueders et al. [31]. Subsequently, the DNA was precipitated using polyethylene glycol 6000 (Sigma Aldrich, Australia), and the DNA pellet was washed once with 70% (v/v) ethanol and resuspended in 50 μL nuclease free water (Qiagen, Australia). DNA concentration and purity were determined by agarose gel electrophoresis and fluorometrically using RiboGreen (Qubit Assay Kit, Invitrogen, Australia) according to the manufacturer’s instructions. The extracted DNA was used as target for quantitative PCR (qPCR), stable isotope probing (SIP) and 16 S rRNA gene sequencing.

### Quantification of archaeal and bacterial 16S rRNA and functional genes

Quantitative PCR was used to determine the abundances of bacteria, archaea, methanogenic archaea and sulfate reducing bacteria in the coal formation water. Standards were prepared using the pGEM®-T Easy Vector System (Promega, Madison, WI, US) and 16 S rRNA PCR products of *Desulfovibrio vulgaris* (DSM 644) and *Methanococcoides burtonii* (DSM 6242, DSMZ Germany). Plasmid inserts were verified by Sanger sequencing (Ramaciotti Centre, UNSW, Australia) using the T7 promotor primer (5’-TAATACGACTCACTATAGGG-3’). DNA targets were measured in four different dilutions of DNA extracts (1:10, 1:50, 1:100, 1:500) and in triplicate. Primer sets specific for the different phylogenetic domains and functional genes were used according to [14, 16]. The qPCR reaction mixtures of 25 μL contained 12.5 μL of the premix solution of the iQ SybrGreen qPCR Kit (Biorad, Hercules, CA, US), 8 μL of PCR-grade water, 1.5 μL of each primer (final concentration 0.28 μM) and 2 μL of template DNA (10 ng). The qPCR was performed using a C1000 Thermal cycler with an CFX96 Real Time System (Biorad, Australia). The specificity of the reaction was confirmed by melting curve analysis and agarose gel electrophoresis to identify unspecific products. Amplification efficiencies for all reactions ranged from 96.3 to 110.5% with an r^2^ value of > 0.99 for standard curve regression. Cell abundances were calculated by accounting for the volume of formation water sampled, the volume eluted after DNA extractions, dilutions prior to qPCR and gene copy numbers.

### QPCR cell abundance normalization

To compensate for the variation in DNA recovery from each sample, qPCR measurements of the 16 S rRNA gene, *mcrA* gene and *dsrA* gene in each sample were normalized with a DNA recovery ratio [32]. The DNA recovery ratio was calculated for each sample by comparing the cell number calculated from the DNA yield against the cell count by microscopy according to Eq. 1.

1 $$R_i = Y_i{\mathrm{/}}\left( {N_i \ast D} \right)$$

Where for each sample *i, R* is the DNA recovery ration, *Y* is the DNA yield measured by Qubit (g/mL), *N* is the microscopy cell count (cells/mL), and *D* is the mean mass of DNA per cell (g). In this study, *D* *=* 7.5 × 10^−15^ g_,_ as calculated from previously published measurements of groundwater samples [33–35]. Each qPCR measurement was then normalized with the respective DNA recovery ratio and gene copy number as per Eq. 2.

2 $$\widehat C_i = C_i{\mathrm{/}}R_i{\mathrm{/}}P_j$$

Where for each sample *i*, *Ĉ* is the normalized abundance (cells/mL), *C* is the qPCR measured abundance (cells/mL), and *R* is the DNA recovery ratio. For each target gene *j*, *P* is the copy number per cell (copies/cell). Average 16 S rRNA copy numbers were used (4.02 for bacteria and 1.63 for archaea according to the rrnDB [36, 37]. The *mcrA* and *dsrA* copy numbers were assumed to be 1 copy per cell.

To calculate the abundance of the different microbial genera involved in methanogenesis and sulfate reduction, qPCR of the *mcrA* and *dsrA* genes were combined with the sequencing data of the archaeal and bacterial 16 S rRNA gene, respectively (Eq. 3).

3 $$A_k = \widetilde A_k \ast \widehat C_i$$

Where for each genus *k*, *A* is the calculated absolute abundance (cells/mL), *Ã* is the relative abundance measured by 16 S rRNA gene sequencing. For each sample *i*, *Ĉ* is the functional gene (*mcrA* or *dsrA*) normalized abundance (cells/mL).

### 16S rRNA gene sequencing

The 16 S rRNA gene of the community DNA from the well sample was sequenced using a 454-FLX sequencer and universal primers 926 F (5’-AAACTYAAAKGAATTGACGG-3’) and 1392 R (5’-ACGGGCGGTGTGRC-3’) for the V6-V8 region as described by Matsuki et al. [38] at the Hawkesbury Institute for the Environment, Sydney, Australia. Sequences were filtered to exclude low-quality reads, primer/barcode regions were trimmed and flowgrams were denoised using MOTHUR (http://www.mothur.org/; [39, 40]). Reads shorter than 250 bp were removed. Chimeras were checked using the UCHIME algorithm [41]. Sequences were classified taxonomically with a confidence threshold of 80% using MOTHUR-formatted SILVA training sets (v.199; https://www.arb-silva.de; [42]).

### DNA-stable isotope probing (SIP)

Coal formation water (90 mL) was transferred anoxically into 120 mL nitrogen degassed serum bottles supplemented with 10 mM ^12^C- or fully ^13^C-labelled acetate (99 atom% ^13^C, Sigma Aldrich, Australia) and incubated in the dark at 20 °C. All incubations were carried out in triplicates and sampled for DNA extraction and stable isotope analyses (DNA-SIP) after 0, 3, and 6 days of incubation. DNA was extracted from 5 mL of culture slurry according to Lueders et al. [31]. Three parallel extractions were carried out, and extracts were pooled for each incubation treatment. DNA was checked by agarose gel electrophoresis and quantified fluorometrically using RiboGreen (Qubit Assay Kit, Invitrogen, Australia) according to the manufacturer’s instructions. Gradient preparation, isopycnic centrifugation, and gradient fractionation were performed as described by Lueders et al. [43]. DNA from each gradient fraction was quantified fluorometrically and by qPCR using archaeal and bacterial primer systems as according to Beckmann et al. [16].

### Community analysis of density fractions

Amplicon libraries were generated from the DNA of the density gradient fractions by following Illumina’s 16 S Metagenomic Sequencing Library Preparation Protocol, using 12.5 ng of template DNA per reaction. PCR cycles for the initial PCR were reduced to 21 to avoid PCR biases from over-amplification. The following universal primer pair was used for the initial amplification, consisting of an Illumina-specific overhang sequence and a locus specific sequence:

926F_Illum(5’TCGTCGGCAGCGTCAGATGTGTATAAGAGACAG[AAACTYAAAKGAATTGRCCG]-3’), 1392R_Illum(5’GTCTCGTGGGCTCGGAGATGTGTATAAGAGACAG[ACGGGCGGTGTGTRC]-3’).

This universal primer pair targets the V6–V8 hypervariable regions of the 16 S Ribosomal RNA gene and has been shown to capture the microbial diversity of bacteria and archaea in a single reaction [44]. PCR products were purified using magnetic bead capture with the Agencourt AMpure XP kit (Beckman Coulter, Australia) and quantified using a fluorometric kit (RiboGreen, Qubit Assay Kit, Invitrogen, Australia) according to the manufacturer’s instructions. Purified amplicons were multiplex sequenced using the MiSeq platform (Ramaciotti Centre for Genomics, UNSW, Australia) according to the manufacturer’s instructions. Paired end sequences were joined using FastqJoin (http://code.google.com/p/ea-utils; Erik [45]). Illumina libraries were quality filtered to truncate reads at positions with Phred scores < Q20 retaining reads > 75 bp and with < 3 low quality bases and no N characters using MOTHUR (http://www.mothur.org/) according to Schloss et al. [39] and Kozich et al. [46]. Chimeras were detected and removed [41] and taxonomy was assigned against the SILVA Database (v.119; https://www.arb-silva.de; [42]).

### Statistical analysis

Statistical analyses of the datasets were carried out according to Clarke [47] and Clarke and Warwick [48], using Primer V6 [49] and XLSTAT (AddinSoft, Paris, France).

## Results

### Biogeochemical characteristics of the western coalfield reservoir

Composition and geochemical characteristics of the coal formation water and the coal from all 4 wells used at the trial site in the Western Coalfields of NSW, Australia were analysed (Table 1, Table S1). The coal was of subbituminous rank [50] and core samples from within the solid seam yielded no detectable microorganisms or quantifiable or PCR amplifiable microbial DNA (Fig. 1d). The formation water had a low salinity (0.013 ± 0.007 ppt) and sulfate concentrations of 265 ± 105 mg/L. No significant sources of N (ammonium, nitrate, nitrite) or P (phosphate) were observed in the formation water (Table S1). The coal formation water was characterized by low biomass of 3.2 × 10^4^ cells/mL (*n* = 5; SD = 1.4 × 10^4^). The Oxidation-Reduction potential (ORP, Ag/AgCl) was suitable for methanogenic activity ranging from −134 to −305 mV with a pH range of 7.8 to 8.7 and a water temperature of 16 °C. Calcium peroxide treatment had a clear impact on the redox potential (Figure S1). The oxic phase of the formation water was characterized by an elevated ORP of −141 ± 82 mV for the first 3 months compared to the untreated and nutrient treated well with an ORP of −253 ± 32 mV and −245 ± 40 mV, respectively. After the removal of CaO_2_, the ORP decreased to −287 ± 66 mV initiating the anoxic phase for the following 15 months.

**Table 1:** Hydrogen vs. carbon isotopic composition of methane (∂^13^C_CH4_ and ∂D_CH4_) produced in coal formation water in all nutrient amendments indicating acetoclastic methane formation

Well amendment	δ^13^C-methane (‰)	δD-methane (‰)
+Nutrients + acetate	−76.9	−280
+Nutrients + calcium peroxide	−54.8	−303
+Nutrients	−56.2	−292

### Methane production and cell concentrations

**Fig. 2:**
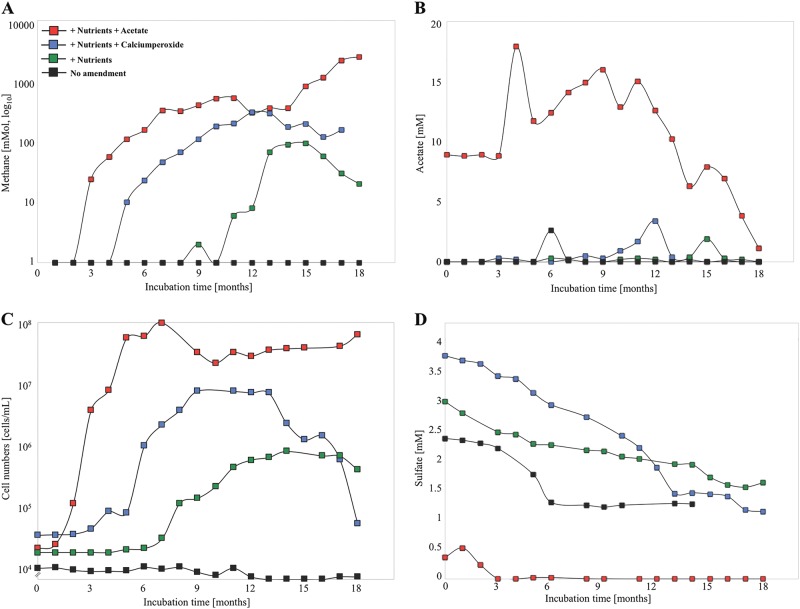
(**a**) Methane formation, (**b**) Acetate concentration, (**c**) Cell numbers, and (**d**) sulfate concentrations in all four *in situ* treatments over an incubation time of 18 months. Addition of nutrients and acetate (red squares), nutrients and calcium peroxide (blue squares), nutrients (green squares) and no amendment (black squares)

Methane production (sum total of methane in the well headspace and formation water) was detected in all nutrient amended wells (N + P, N + P + CaO_2_, N + P + Acetate), showing a distinct one or two phase exponential increase in methane concentration (Fig. 2a). The highest methane yields were observed in a positive control well amended with the methanogenic substrate acetate (Fig. 2a, b). Acetate (final concentration 10 mM) was added at the start of the field trial and after 3 months to a final concentration of 20 mM in the coal formation water. Two phases were apparent in early biomass proliferation up to 7 months with a 3.5 order of magnitude increase in cell numbers, then declining almost 1 order of magnitude for 2 months until a second proliferation from 10 to 18 months (Fig. 2c). Both phases of methane generation were linked to acetate consumption (Fig. 2b).

The second highest methane formation yields were observed in the nutrient plus CaO_2_ amended well (Fig. 2a). After the removal of CaO_2_, methane increased over 5-fold peaking at 12 months. Cell concentrations started to increase after the removal of the CaO_2_ from 10^5^ to 10^7^ cells/mL and decreased with the decline of methane (Fig. 2a, c). In the well amended with nutrients only, methane increased from 12–15 months (Fig. 2a) with an 10-fold increase in cell numbers (Fig. 2c). The stable carbon and hydrogen isotopic analyses of the methane in all wells exhibited light carbon isotopic signatures with ∂^13^C values ranging from −55 to −77 ‰ relative to VPDB (Vienna Peedee Belemnite) indicating a biogenic origin of methane and with ∂D-CH_4_ of −300 ‰ consistent with acetoclastic methanogenesis ([11, 28]; Table 1) indicating that acetate was the main precursor of methane formation in all treatments.

### Microbial community composition in the coal formation water

**Fig. 3:**
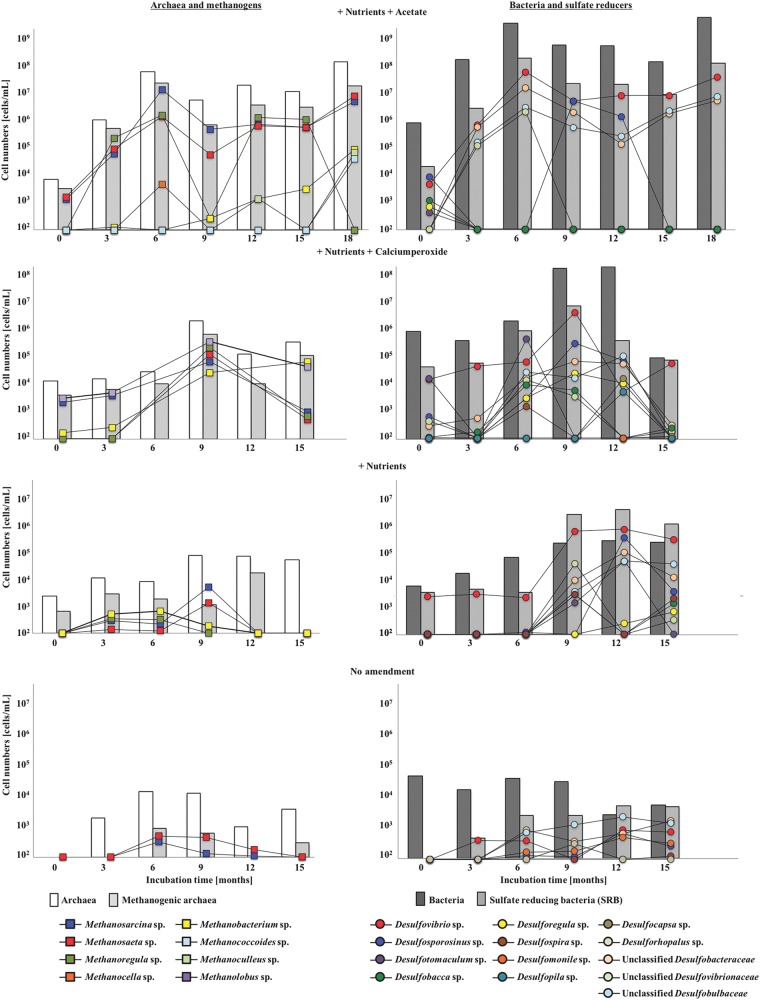
Cell numbers of archaea (white bars), methanogenic archaea (light grey bars), bacteria (dark grey bars) and sulfate reducing bacteria (grey bars) based on ribosomal RNA and the functional *mcrA* and *dsrA* genes determined by quantitative qPCR. Changes of absolute abundances of methanogens (coloured squares) and sulfate reducing bacteria (coloured circles) over an incubation time of 15–18 months

DNA extraction from the coal formation water was performed every three months and DNA subjected to PCR amplification, 16S rRNA gene sequencing and quantitative PCR of 16S rRNA and functional genes (*mcrA* and *dsrA*) to determine the microbial community composition and abundance in all wells over an incubation time of 15–18 months. In addition, qPCR copy numbers were normalised to total cell counts as determined by microscopy. All nutrient amended well treatments altered the archaeal and bacterial community composition significantly in the coal formation water in favour of methane production, directly by methanogens and indirectly by potential hydrocarbon degrading bacteria, especially sulfate reducing bacteria (Figs. 2a, 3).

**Fig. 4:**
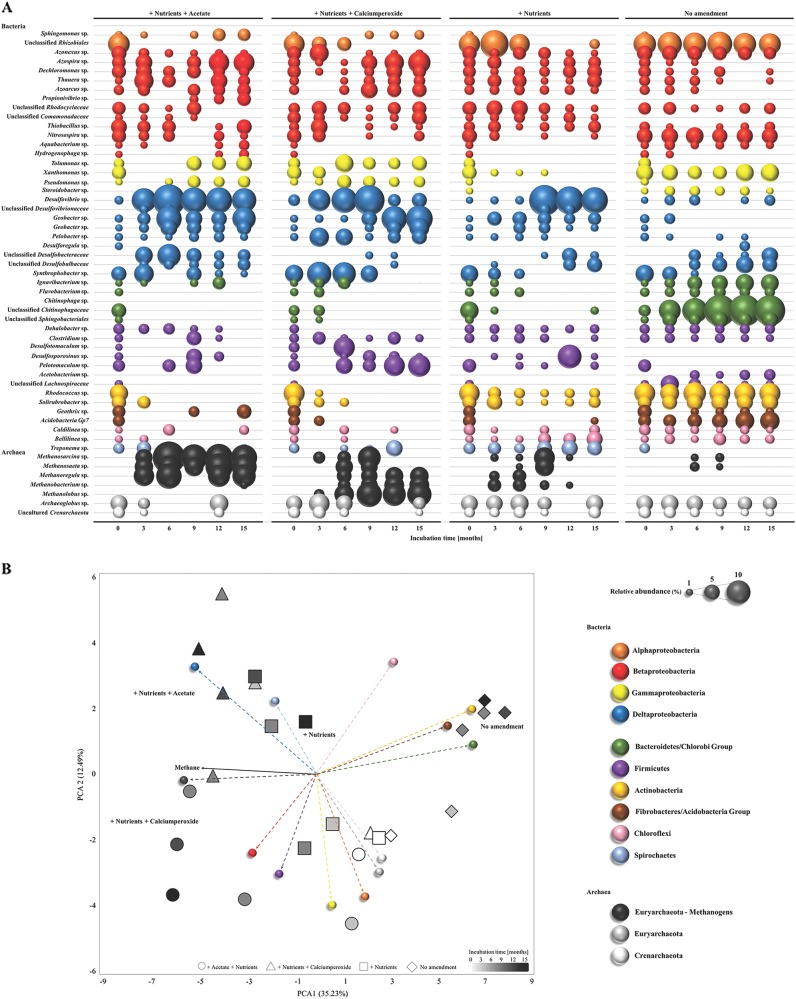
**a** Changes in the bacterial and archaeal community composition in the coal formation water in response to different nutrient amendments over an incubation time of 15 months based on 16 S rRNA gene sequencing. From left to right: nutrients and acetate (positive control), nutrients and calcium peroxide, nutrients and no amendment (negative control). Colors indicate members of different phyla. Size of the bubble represents relative abundance of the genus or family in each sample. **b** Principal component analysis (PCA) comparing four different wells amended with nutrients and acetate (triangles), nutrients and calcium peroxide (circles), nutrients (squares) and no amendment (diamonds) over an incubation time of 15 months (white = 0 months to black = 15 months) and the showing the relationship to the different phyla (Bubbles are color coded as in **a** and methane production

In response to the nutrient amendments, methanogenic archaea increased from < 1 to up to 20% of the microbial community composition based on 16S rRNA gene sequencing and *mcrA* gene qPCR data (Figs. 3, 4a). Methanogenic archaea detected in the coal formation water belonged to the orders *Methanosarcinales* (*Methanosarcina* sp., *Methanosaeta* sp., *Methanolobus* sp. *Methanococcoides* sp.), *Methanomicrobiales* (*Methanoregula* sp., *Methanoculleus* sp.) and *Methanobacteriales* (*Methanobacterium* sp.) and their abundance increased significantly up to 3-4 orders of magnitude in the nutrient amended wells with an initial oxic phase or the addition of acetate (10^6^ and 10^7^ cells/mL respectively; *T*-test, *p* *=* 0.023). The genera *Methanosarcina*, *Methanosaeta*, *Methanoregula* and *Methanolobus* were principally responsible for this increase with *Methanobacterium*, *Methanococcoides* and *Methanoculleus* species contributing to a later methane production phase (Fig. 3). Low methanogenic cell numbers ( < 10^3^ cells/mL) were observed in the untreated control well congruent with infrequent detection of *Methanosaeta* and *Methanosarcina* species (Figs. 3, 4).

The nutrient-stimulated microbial communities were significantly distinct from the untreated control (ANOSIM, *p* < 0.003) that harboured the indigenous community of the coal formation water. The indigenous community was dominated by bacterial members of the Beta- and Deltaproteobacteria, the Bacteroidetes/Chlorobi Group, the Firmicutes, the Actinobacteria and Fibrobacteres/Acidobacteria Group (Fig. 4a, b). In nutrient amended wells a decrease in relative abundance of members affiliated with the Bacteroidetes/Chlorobi group (mainly Unclassified *Chitinophagaceae*; explaining 19.16–28.08% of the dissimilarity compared to the untreated control well; SIMPER analysis) and the Actinobacteria (mainly *Rhodococcus* sp.; 9.46–10.02% dissimilarity; SIMPER analysis) was observed along with an increase in Deltaproteobacteria (mainly *Desulfovibrio*, *Geobacter, Syntrophobacter, Pelobacter* sp.; 16.18–18.93% of the dissimilarity; SIMPER analysis) and methanogenic archaea (mainly *Methanosarcina*, *Methanosaeta*, *Methanoregula*, *Methanolobus*, *Methanobacterium* sp.; 12.34–21.32% of the dissimilarity, SIMPER analysis). A higher dissimilarity of the microbial communities was observed between the untreated well and the nutrient plus acetate treated well (72.89% dissimilarity, SIMPER analysis) or the nutrient and calcium peroxide amended well (71.32% dissimilarity, SIMPER analysis). In comparison, the dissimilarity between the untreated well and the well only amended with nutrients (56.62% dissimilarity, SIMPER analysis) was lower (Fig. 4b). Nutrient amendments with acetate or calcium peroxide resulted in significantly different microbial community compositions compared to the nutrient amended well (ANOSIM, *p* = 0.029 and *p* = 0.034) whereas communities amended with acetate and calcium peroxide were not significantly different (ANOSIM, *p* = 0.07).

Sulfate reducing lineages, mainly Deltaproteobacteria and *Archaeoglobales* were detected in all wells and increased in abundance along with the *dsr*A gene (10^4^ to 10^8^ cells/mL) in the nutrient amended wells throughout the field trial (Fig. 3, 4a). Highest SRB abundance and diversity were observed in the acetate plus nutrient amended well compared to the well only amended with nutrients (Fig. 3). The sulfate reducing communities were dominated by *Desulfovibrio* spp. and members of the *Desulfobacteraceae*, *Desulfovibrionaeceae* and *Desulfobulbaceae* families (Fig. 3). The calcium peroxide treatment resulted in a decrease in SRB abundance and diversity (*Desulfovibrio* sp. being an exception) but recovered when calcium peroxide was removed and the anoxic incubation phase initiated (Figs. 3, 4a).

### Microbial community composition on the coal surface

**Fig. 5:**
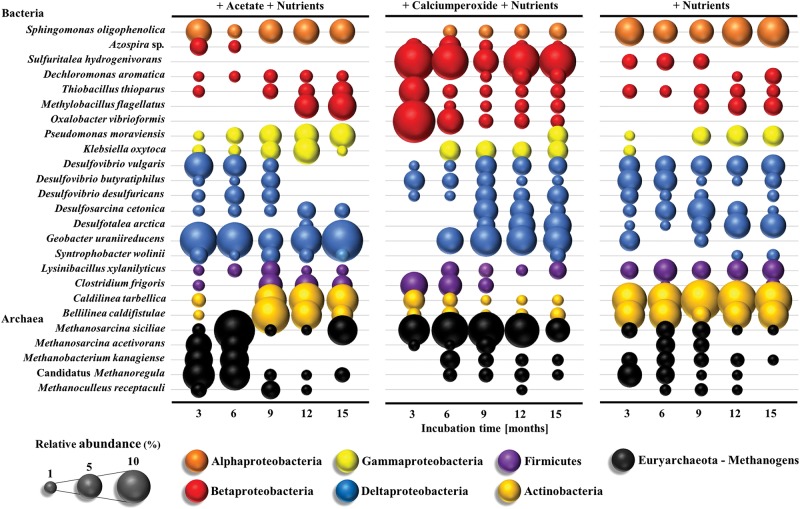
Changes in the bacterial and archaeal community composition on the coal surface in response to different nutrient amendments over an incubation time of 15 months based on 16 S rRNA gene sequencing. From left to right: nutrients and acetate (positive control), nutrients and calcium peroxide, nutrients and no amendment (negative control). Colours indicate members of different phyla. Size of the bubble represents relative abundance of the genus or family in each sample

In response to the nutrient amendments, methanogenic archaea as well as bacteria were detected at the surface of the subbituminous coal after 3 months of the field trial (Fig. 5). The coal obtained from the untreated well and at the start of the field trial yielded no detectable microorganisms or microbial DNA. After 3 months of incubation, methanogenic archaea were detected at the coal surface of all amendments and belonged to the orders *Methanosarcinales* (*Methanosarcina* sp.), *Methanobacteriales* (*Methanobacterium* sp.) and *Methanomicrobiales* (Candidatus *Methanoregula*, *Methanoculleus* sp.). Their relative abundance increased from 0 to 37% after the addition of acetate and from 0 to 19% after the calcium peroxide induced oxic phase ceased compared to an increase from 0 to 12% when only amended with nutrients. Bacterial lineages within the phyla Alpha-Deltaproteobacteria, Actinobacteria and Firmicutes associated with sulfate- and sulfur reduction and hydrocarbon oxidation predominated.

Sulfate reducing bacteria (SRB) within the orders *Desulfovibrionales* and *Desulfobacterales* increased in relative abundance after the addition of acetate (0 to 11%) and in the calcium peroxide treatment (0 to 19%) after the anoxic phase was initiated. Both treatments showed a high relative abundance of *Geobacter uraniireducens* (up to 14%) attached to the coal surface.

### Microbial metabolic potential

**Fig. 6:**
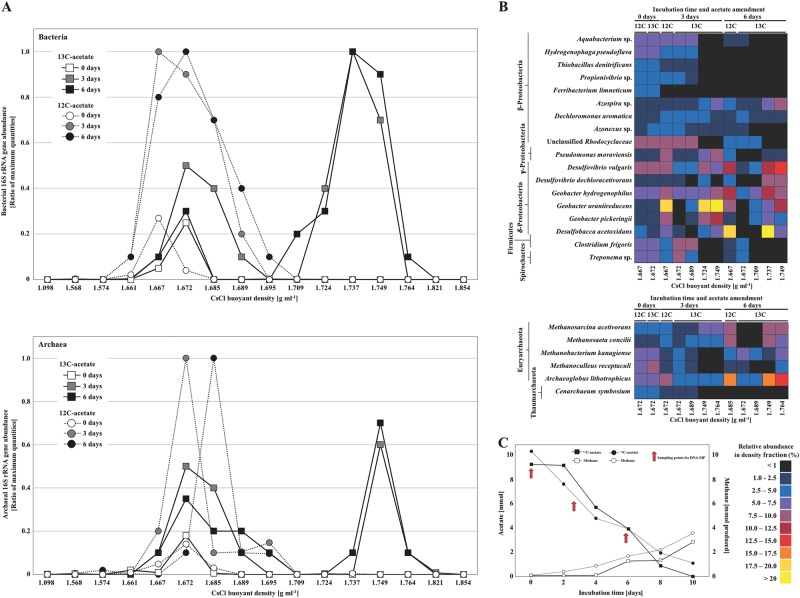
**a**, **b** Distribution and relative abundances of bacteria and archaea significantly associated with acetate utilisation in the ‘heavy’ and ‘light’ fractions of CsCl gradients derived from samples incubated in the presence of either ^13^C-labelled acetate or ^12^C-labelled acetate. **c** Acetate consumption and methane production in ^13^C- and ^12^C-labelled acetate amendments of coal formation water from the nutrient and acetate amended well (^13^C-acetate = black squares, ^12^C-acetate = black circles, ^13^C-methane = white squares, ^12^C-methane = white circles). DNA samples were taken from the ^13^C- and ^12^C-acetate enrichments during the time course of acetate consumption (red circles)

DNA stable isotope probing (DNA-SIP) was used to track the incorporation of the methanogenic substrate acetate by bacteria and archaea in the coal formation water of the nutrient and acetate amended well (Fig. 6). Formation water sampled at the end of the field trial (18 months; Fig. 2a) was incubated with ^13^C_2_-acetate for 10 days. Labelled and unlabelled acetate pulses (10 mM) were consumed in 10 days (Fig. 6c). Methane increased after 4 days of incubation and continued to accumulate up to the conclusion of the incubation (Fig. 6c).

SSU 16S rRNA gene sequences from gradient fractions ranging in buoyant density from heavily labelled (1.764 g mL^−1^) to unlabelled (1.667 g mL^−1^) were compared within ^13^C-acetate-incubated samples and ^12^C-acetate-incubated controls (Fig. 6a). After 3 and 6 days, strong ^13^C dependent community shifts within the ‘light’ and ‘heavy’ gradient fractions were observed. Although many species were detected in both the ‘heavy’ and the ‘light’ gradient fractions, there was a clear distribution trend in the relative abundance of species (Fig. 6b). The early incorporation of ^13^C-label was predominantly detected in bacteria closely related to *Geobacter uraniireducens*, *Geobacter pickeringii*, *Geobacter hydrogenophilus*, *Desulfovibrio vulgaris* and *Desulfobacca acetoxidans* (Deltaproteobacteria) after day 3 in response to labelled acetate exposure (Fig. 6a, b). Organisms that incorporated carbon from acetate during the time course of 6 days belonged to the *Azospira* sp., *Pseudomonas moraviensis* (Betaproteobacteria) and *Desulfovibrio dechloracetivorans* (Deltaproteobacteria). Acetoclastic methanogenic archaea related to *Methanosarcina acetivorans* and *Methanosaeta concilii* incorporated ^13^C-acetate after 6 days when 65% acetate was consumed (Fig. 6b, c). Additionally, a non-methanogenic member within the phylum Euryarchaeota affiliated to *Archaeoglobus lithotrophicus* became highly enriched in the ‘heavy’ DNA fraction after 6 days (Fig. 6b). *Archaeoglobus* species are known as hyperthermophilic archaea found in high-temperature oil reservoirs potentially mediating sulfate reduction and hydrocarbon degradation [51–53]. Recently 16S rRNA sequences similar to those of *Archaeaoglobus* species were detected in two moderate-temperature coal reservoirs [54, 55].

## Discussion

This study is the first to observe long-term (1.5 years) succession of microbial communities in a coal seam in response to biostimulation for biogas production. It was conducted in a gas-free sulfate-rich subbituminous coal seam resulting in confidence that all gas produced resulted from the stimulatory amendments applied and that biogas production is possible in the presence of significant quantities of sulfate. Basic nutrients (ammonium and phosphate) and the oxygen releasing reagent calcium peroxide (CaO_2_) were used for the first time to stimulate methane production.

### Stimulation of multiple microbiomes in a single coal seam

We present a pragmatic longitudinal study in which four individual wells were amended with nutrients, nutrients with calcium peroxide, nutrients with acetate (positive control well) or not amended (negative control well) and responses monitored over a period of 15–18 months. Given the sampling and access constraints to gas production wells in general, this study represented a valuable opportunity to assess succession in the microbial communities over a long-term stimulation trial. Whilst there were some differences in starting conditions in each well (eg. variation in sulfate and chloride concentrations), pH and ORP, generally considered major drivers of community composition, were uniform. Regardless of initial differences, quantitative PCR of 16S rRNA and functional genes (*mcrA*, *dsrA*), 16S rRNA sequencing and geochemistry analysis of the coal formation water revealed that the microbiomes of the different wells changed in relation to treatments (Figs. 3, 4). Depending on the availability of nutrients and the presence of oxic or anoxic conditions, the microbial communities were dominated by members of the Beta- and Deltaproteobacteria, Firmicutes and methanogenic Euryarchaeota (Fig. 4). The metabolic capacities of these microbial communities were respectively centred either on oxidation of coal hydrocarbons, acetogenesis, sulfate reduction, iron reduction and methane formation. These findings may have important implications for gas enterprises to operate extraction wells and stimulate biogas formation.

### Formation water chemistry dictates the indigenous microbial community composition

The formation water composition was a major factor shaping the composition and metabolic potential of the initial reservoir microbiome (Figs. 1, 3, 4 Table S1). Sequencing and qPCR of functional genes (*mcrA* and *dsrA*) indicated a strong potential for sulfate reduction and a very low potential for methanogenesis (methanogenic archaea; Figs. 3, 4a). The initial archaeal community composition was similar in all wells, with a predominance of *Archaeoglobus* sp. and uncultured Crenarchaeota (Fig. 4a), commonly observed in coal and oil reservoirs worldwide [51, 53, 55–59]. The considerable potential of *Archaeoglobus* species to ferment alkanes, a main constituent of coal and oil, to generate acetate has been shown recently [58]. The produced acetate could then provide a substrate source for methanogens as well as sulfate reducers. *Archaeoglobus* species also have the capacity to couple the degradation of hydrocarbons, like alkanes to sulfate reduction [53, 56, 60]. Metagenomic mining, genomic bin reconstruction and functional gene sequencing (*dsrAB* genes) indicated a strong potential for sulfate reduction and hydrogen sulphide production. Metagenomic bins contained the *assA*-gene, which encodes the catalytic subunit of an alkylsuccinate synthase, an enzyme for the activation of hydrocarbons by fumarate addition and genes for the utilization of activated hydrocarbons as well as genes involved in β-oxidation and the utilization of acetyl CoA [58]. Similar pathways have been identified in isolates [61] but the exact mechanism of hydrocarbon degradation is still unclear.

Crenarchaeota and closely related members of the Thaumarchaeota and Miscellaneous Crenarchaeotal Group (MCG) have been shown to carry out a heterotrophic metabolism rather than being obligate autotrophic. They can use organic carbon derived from the degradation of fossil organic matter and are likely to access substrates that are physically or chemically recalcitrant, utilizing organic compounds found in wastewater and petroleum reservoirs [62–66] . Crenarchaeota have been shown to dominate sulfate-reducing enrichments from petroleum reservoirs with a pronounced degradation of alkanes where the anaerobic activation of alkanes proceeded potentially via *assA*-genes, or alternative yet unknown mechanisms [67].

Members of Archaeoglobus and Crenarchaeota might potentially be involved in the breakdown of coal-associated hydrocarbon compounds like alkanes and fatty acids to acetate using sulfate as electron acceptor. One important characteristic of the coal formation water was the presence of moderate to high sulfate concentrations (0.5 to 3.7 mM sulfate; Fig. 2d) creating an environment where the methanogenic archaea might be outcompeted by SRB for acetate and H_2_ gas [26]. Elevated concentrations of sulfate have been observed in coalbed aquifers, but not in association with methane formation [24, 68]. Together this indicates that in elevated-sulfate, and low-nutrient formation waters, indigenous microbial communities were potentially linked to hydrocarbon oxidation coupled to sulfate reduction rather than to methanogenesis.

### The rise of hydrocarbon degrading bacteria through the addition of nutrients

After nutrient treatments were applied, hydrocarbon degrading bacteria became abundant, affiliated with *Azospira*, *Dechloromonas*, *Thauera*, *Azoarcus*, *Propionivibrio* (Betaproteobacteria), *Tolumonas, Pseudomonas* (Gammaproteobacteria), *Desulfovibrio*, *Geobacter*, *Pelobacter*, *Synthrophobacter* (*Deltaproteobacteria*), *Desulfotomaculum, Desulfosporosinus* and *Pelotomaculum* species (Firmicutes; Fig. 4a). These lineages have a cosmopolitan distribution with their presence previously noted in coalbed seams worldwide, e.g. Powder River Basin, Ruhr Basin, Alberta Basin, Illinois Basin, Ishikari Basin, Waikato coalfields, deep sea coalbeds [14, 16, 21, 57, 59, 69–71]. Whereas the most dominant bacterial phyla were represented by the Firmicutes, Spirochaetes, Bacteroidetes and all five groups of the Proteobacteria [5]. Their predominance can be associated with the solubilisation of intermediates from coal using a range of genes (*bssA*, *bamA*, *bamB*, *bzdN*, *bcrA*, *bcrC)* involved in the degradation of a variety of hydrocarbons being capable of initially attacking and metabolizing aromatic hydrocarbons [72–74]. Specifically, *Thauera*, *Azoarcus*, *Pseudomonas*, and *Geobacter* species as well as SRB are recognized as key organisms for BTEX (Benzene, Toluene, Ethylbenzene, Xylene) and/or PAH (Polycyclic Aromatic Hydrocarbons) degradation under nitrate reducing, iron and sulfate reducing conditions [73, 75–79]. The potential utilization of different electron acceptors by hydrocarbon degrading bacteria suggests a high metabolic versatility of the microbial community which could respond more easily to changes in their availability and with the degradation of coal regardless of the electron acceptor available as observed in other coal habitats [57]. Another major group involved in anaerobic aromatic hydrocarbon degradation is the *Peptococcaceae* (Firmicutes) such as *Desulfotomaculum*, *Desulfosporosinus* and *Pelotomaculum* species (Fig. 4a; [72, 73]). These species were abundant in the calcium peroxide and nutrient amended well after the initial oxic conditions ceased and anoxic conditions were induced (Fig. 4a). Members of the phylum Firmicutes, potentially using methanol and methylamines as the sole carbon and energy source, dominate coal-bearing sediments in the deep sea [71, 80]. Congruently, *Pelobacter* and *Syntrophobacter* species increased in relative abundance (Fig. 4a) suggesting the initial oxic phase stimulated subsequent anaerobic hydrocarbon degrading processes through release of aliphatic and aromatic compounds. Additionally, acetogenic bacteria putatively involved in hydrocarbon degradation were observed in the formation water (*Clostridium*, *Dechloromonas;* Fig. 4a). Notably, sequences similar to *Clostridiu*m species were observed in earlier surveys in coal habitats [14, 16, 59, 81]. All the putative hydrocarbon-degrading bacteria may be involved in the initial breakdown of coal and its transformation into compounds further utilised by the remainder of the microbial community. During the long term incubations the accumulation of acetate was detected occasionally when methane production stalled (Fig. 2a, b) implying that the activity of acetogenic bacteria provided acetate to the methanogenic archaea.

### Acetate is the central energy carrier responsible for methane production

Increases in abundance of methanogenic archaea and biogenic methane formation were stimulated in all nutrient amended wells (Figs. 2a, 3, 4a, b). Here we demonstrate that nutrient amendment results in the direct or indirect (via acetogenic bacteria) stimulation of methanogenic archaea, mainly *Methanosaeta* spp. and *Methanosarcina* spp., which are obligate and facultative acetoclastic methanogenic archaea respectively (Figs. 3, 4a). Compound specific isotope analyses of methane indicated acetoclastic methanogensis to be the dominant pathway for methane production (Table 1). Recent studies have shown that in organic-matter rich sediments, acetate was not directly converted to methane but rather oxidised to CO_2_ in a syntrophic relationship between acetotrophic bacteria and methanogens favouring hydrogenotrophic methanogenesis [82]. Hydrogenotrophic methanogenesis has been detected in many coal reservoirs predominantly under sulfate-limiting conditions [5, 71]. However, in the coal formation water, hydrogenotrophic methanogens appeared to be more abundant during the late methane production phase or the ultimate plateau when more biomass generated through biostimulation was likely hydrolysed and fermented (Figs. 2a, 3). H_2_ was not detected, most likely due to fast microbial utilization. Our results indicate that adding the methanogenic substrate acetate to prime the microbial system for enhanced methane production is not efficient since methane production accounted for only 28% of the acetate added.

A promising carbon independent amendment approach is the application of oxygen releasing agents such as calcium peroxide that lifts the ORP and potentially stimulates the initial oxidative biofragmentation of coal. This would result in the release of aliphatic and aromatic compounds that can be taken up by cells and fermented to yield electron donors such as acetate for methanogenic archaea. Microbial uptake of oxygen creates anaerobic microenvironments where the methanogenic archaea are protected, however oxidative stress strongly inhibits methanogenesis [83]. *Methanosarcina* and *Methanolobus* sp. were abundant during the first 3 months of incubation (Fig. 3), when the ORP was high (Figure S1), indicating their resilience to extended exposure to microaerophilic conditions and involvement in the initial methane production. Though methanogenic archaea are regarded as strictly anaerobic, some methanogenic archaea (eg. *Methanosarcina*) can tolerate prolonged periods of oxygen exposure [84, 85]. The abundance of obligate acetoclastic methanogenic genera, specifically *Methanosaeta* sp., increased after the removal of CaO_2_, potentially preferring a more reduced environment (Fig. 3).

### The fate of acetate in the coal formation water

While 28% of acetate in the acetate amended positive-control well was converted to methane *in situ*, the fate of the remaining acetate was unclear (Fig. 2a, b). DNA stable isotope probing (DNA-SIP) was used to track the incorporation of acetate by bacteria and archaea in the coal formation water of the nutrient and acetate amended well.

Elevated sulfate concentrations and high relative abundances of specialised acetate oxidising bacteria, such as *Desulfobacca acetoxidans* [86], indicates a potential competition between sulfate reducing bacteria and acetoclastic methanogenic archaea, as described in various anoxic systems [26, 87, 88]. There was no increase in SRB abundance in the well amended with nutrients only (Fig. 3) indicating that acetate was the main stimulant for SRB. Intermittent acetate production with up to 4 mM acetate was observed in all nutrient amendments but otherwise the acetate concentration was below the detection limit of 50 μM (Fig. 2). Acetogenesis from H_2_ and CO_2_ would be possible below a concentration < 100 μM [89].

H_2_ was not detected in the coal formation water during the field trial assuming concentrations below the detection limit of 100 μM. Both, H_2_ and acetate, produced via bacterial fermentation of complex organic matter [90, 91], are key substrates to methanogens and SRB [87], and a rapid turnover results in acetate and H_2_ typically not accumulating to high concentrations, unlike the end products of sulfate reduction (H_2_S) and methanogenesis (CH_4_; [92, 93]).

Methanogenesis and sulfate reduction, using H_2_ as electron donor, were still thermodynamically favourable at the detection limit concentrations of 100 μM with energy yields exceeding the estimated biological energy quantum (BEQ) of ΔGr = −10 kJ mol^−1^ [89]. If H_2_ is the energy source, methanogens and SRB can support a H_2_ driven metabolism with energy yields as low as −10 and −20 kJ mol^−1^, respectively [89, 94]. Energy yields (ΔGr) for sulfate reduction and methanogenesis from H_2_ as electron donor, would be −130 and −90 kJ mol^−1^, for a detection limit of 100 μM H_2_ [89] with a higher free energy yield for SRB. Under H_2_ limiting conditions, methanogens can still gain energy down to a H_2_ concentration of 11 nM, whilst sulfate reducing bacteria can meet the BEQ down to a H_2_ concentration of 0.6 nM [89]. At an acetate concentration of 50 μM, the dG’ for acetate driven sulfate reduction would be −22.8 kJ mol^−1^ and for methanogenesis −6.5 kJ mol^−1^, suggesting sulfate reducers would outcompete methanogens for acetate.

SRB are usually obtained in higher cell densities and with higher turnover rates compared to methanogens, excluding methanogens from the consumption of acetate. SRB are capable of carrying out sulfate reduction using acetate concentrations as low as 1–20 μM. In the acetate amended well, the relative abundance of SRB was higher (10^6^–10^8^ cells/mL) than needed for the sulfate reduction observed in the later stage of the field trial where only 2 × 10^5^ cells/mL would be sufficient for the reduction of 2.5–4 mM sulfate (Figs. 2, 3), indicating the relevance of potential electron acceptors other than sulfate, e.g. iron (III). Many SRB are capable of oxidizing acetate and reducing iron (III) [86, 95, 96]. SRB reduction rates were slow and the addition of nutrients did not lead to an exhaustion of the sulfate in the coal formation water during the 18 month field trial (Fig. 2), except in response to exogenous addition of acetate supporting the fact that SRB are energy (acetate) limited.

The early incorporation of ^13^C-label was predominantly into bacteria closely related to *Geobacter uraniireducens*, *Geobacter pickeringii*, *Geobacter hydrogenophilus*, *Desulfovibrio vulgaris* and *Desulfobacca acetoxidans* (Deltaproteobacteria) after day 3 in response to acetate exposure suggesting that these species are responsible for acetate degradation (Fig. 5a, b). The strong response of *Geobacter* species capable of acetate oxidation and Fe(III) reduction [97, 98] suggests that Fe(III) reduction might play an important role in acetate metabolism in coal formation water potentially influenced by iron well casing. Members of the genus *Geobacter* are often the predominant Fe(III)-reducing bacteria in subsurface anoxic environments [98]. It has been hypothesized that *Desulfovibrio* species may also play an important role in the reduction of Fe(III) [95, 99, 100]. Iron (III) was the only electron acceptor detected in the formation water at a concentration of 0.5 mM accounting for only 0.06 mM acetate oxidised to carbon dioxide regarding stoichiometric conversions suggesting that Fe(III) is potentially used from the sediment at the bottom of the well or from the iron well casing representing another potential source of Fe(III). Acetoclastic methanogenic archaea related to *Methanosarcina acetivorans* and *Methanosaeta concilii* incorporated ^13^C-acetate after 6 days when 65% of acetate was already consumed (Fig. 5b), possibly due to a slower response to acetate exposure alongside the abundant *Geobacter* species. The *ex situ* methane yield obtained supports our findings *in* *situ* that methane is not the major sink of acetate, with only 30–40% used by methanogenesis (Fig. 6c). Most notably, sulfate- and iron-reducing bacteria (*Desulfovibrio* and *Geobacter*) compete effectively for acetate.

### The coal surface: a stage for interspecies interactions?

Many studies have elucidated the composition of microbial communities in coal formation water, but the presence of methanogenic archaea, sulfate reducing bacteria and hydrocarbon degrading bacteria on the coal surface *in situ* or over a long-term field trial has not previously been investigated. It has been hypothesised that hydrocarbon oxidising and fermentative bacteria occupy the coal surface and perform the first steps of coal degradation whilst methanogenic archaea use solublised reducing equivalents in the coal formation water to produce methane [5]. In conflict with this hypothesis we have shown that methanogenic archaea make up a crucial part of the microbial community on the coal surface (Fig. 5). These findings are supported by recent investigations by Trembath-Reichert et al. [80] and Inagaki et al. [71] showing that microbial growth is possible within a deep subseafloor coal bed, predominated by sulfate reducing and hydrocarbon degrading bacteria utilizing mainly methylated substrates and hydrogenotrophic methanogens being responsible for the vast majority of biogenic methane formation. All nutrient amendments in the long-term field trial resulted in the colonization of the coal surface by methanogenic archaea, sulfate reducing bacteria and hydrocarbon degrading bacteria (Fig. 5). No microorganisms were detected on the coal surface in the unamended control well. This is congruent with the idea that cell increase and methane production from coal is limited by the lack of available nutrients [101]. After the oxic phase ceased, the predominance of anaerobic hydrocarbon degraders and methanogenic archaea on the coal surface suggests enhanced degradation of available coal compounds to methanogenic substrates.

## Conclusion

**Fig. 7:**
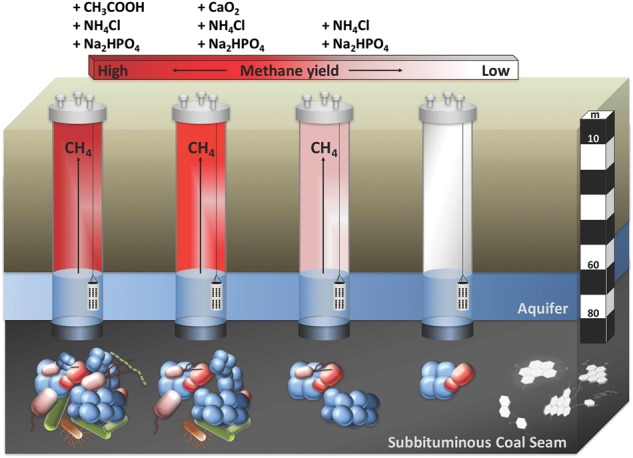
Long-term microbial community succession in coal formation water biostimulated for enhanced methanogenesis was described. High sulfate concentrations and SRB did not prevent the stimulation of methanogenic archaea that are nutrient limited through the addition of a mineral nutrient amendment. Methanogenic archaea make up a major part of the coal associated biofilm communities carrying out acetoclastic methanogenesis in a gas-free coal seam. Acetate, and not H_2,_ is the central energy carrier in this coal reservoir. SRB as well as iron reducing bacteria (IRB) are energy (acetate) limited and compete with the methanogenic archaea for acetate. Only 28% of the amended acetate accounted for methane production, the majority was consumed by SRB and IRB

Long-term microbial community succession in coal formation water biostimulated for enhanced methanogenesis was described (Fig. 7). High sulfate concentrations and SRB did not prevent the stimulation of methanogenic archaea that are nutrient limited through the addition of a mineral nutrient amendment. Methanogenic archaea make up a major part of the coal associated biofilm communities carrying out acetoclastic methanogenesis in a gas-free coal seam. Acetate, and not H_2,_ is the central energy carrier in this coal reservoir. SRB as well as iron reducing bacteria (IRB) are energy (acetate) limited and compete with the methanogenic archaea for acetate. Only 28% of the amended acetate accounted for methane production, the majority was consumed by SRB and IRB.

## Electronic supplementary material

Supplementary material
