# Supplementary material for: Long-term succession in a coal seam microbiome during in situ biostimulation of coalbed-methane generation
Source: ISME J. 2018 Oct 15;13(3):632–50. doi: 10.1038/s41396-018-0296-5 (PMC6461797; doi:10.1038/s41396-018-0296-5)
Supplement: Supplementary file 1 — Supplementary material [file 41396_2018_296_MOESM1_ESM.docx]

**Supplementary Material**

**Supplementary Table 1.** Chemical composition (mg/L) of the coal formation water in the coal gas wells 1-4. SD = Standard deviation. ND = Not detected. IDL = Instrument detection limit.

**Supplementary Table 2.** Petrographic parameters and chemical composition of the subbituminous coal by mass percentage.

**Supplementary Figure 1.** (A) Oxidation-Reduction Potential (ORP; Ag/AgCl), (B) ammonium concentration, (C) nitrite, and (D) nitrate concentrations in all four *in situ* treatments over an incubation time of 18 months. Addition of nutrients and acetate (red squares), nutrients and calcium peroxide (blue squares), nutrients (green squares) and no amendment (black squares).

**Supplementary Figure 2.** *Ex situ* methane production in coal formation water amended with coal [black triangles and squares] and iron (II) [triangles] over an incubation period of 12 months. Methane can be generated through the reaction of iron with groundwater releasing H_2_ that can be used by acetogenic bacteria or hydrogenotrophic methanogenic archaea. The cultures were set up according to *in situ* proportions of coal and iron (well casing) surface area to groundwater volume and incubated over a period of 12 months. Highest methane production rates were still detected in the cultures amended with iron and coal (SF 1) supporting the potential contribution of well casing to the overall methane formation budget. Nevertheless, the majority of methane is derived from the coal over this period.

**Supplementary Table 1.**

**
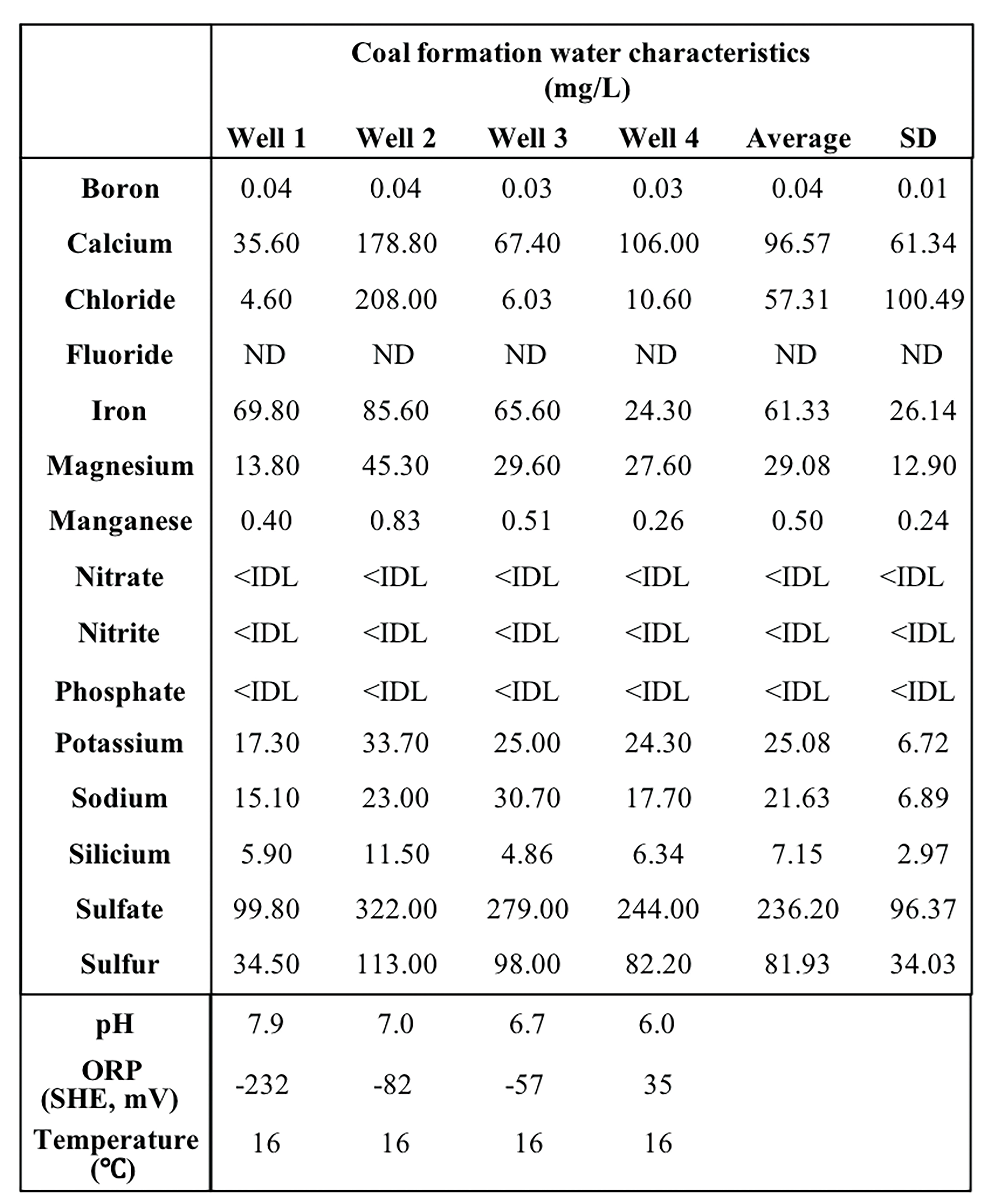
**

**Supplementary Table 2.**

**
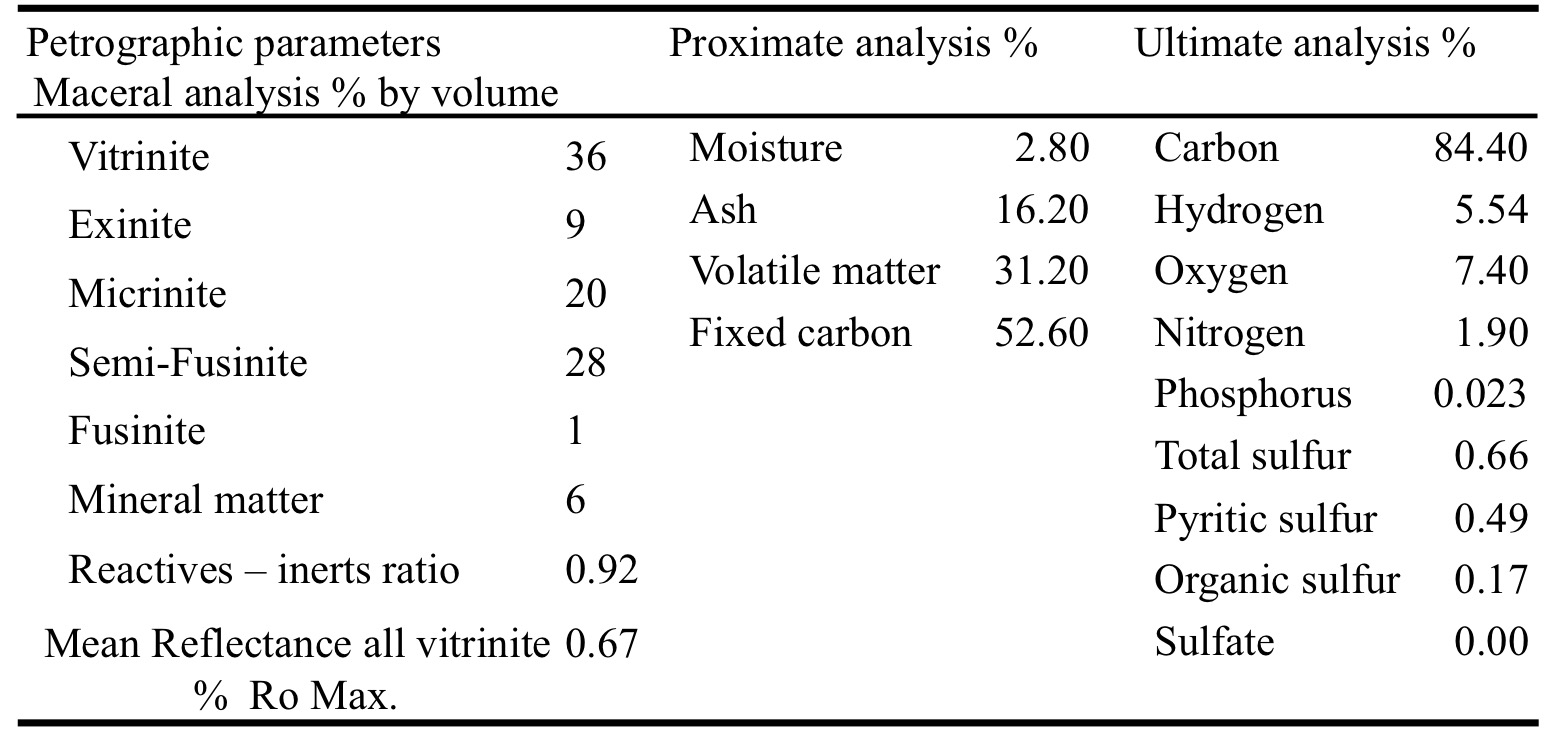
**

**Supplementary Figure 1.**

**
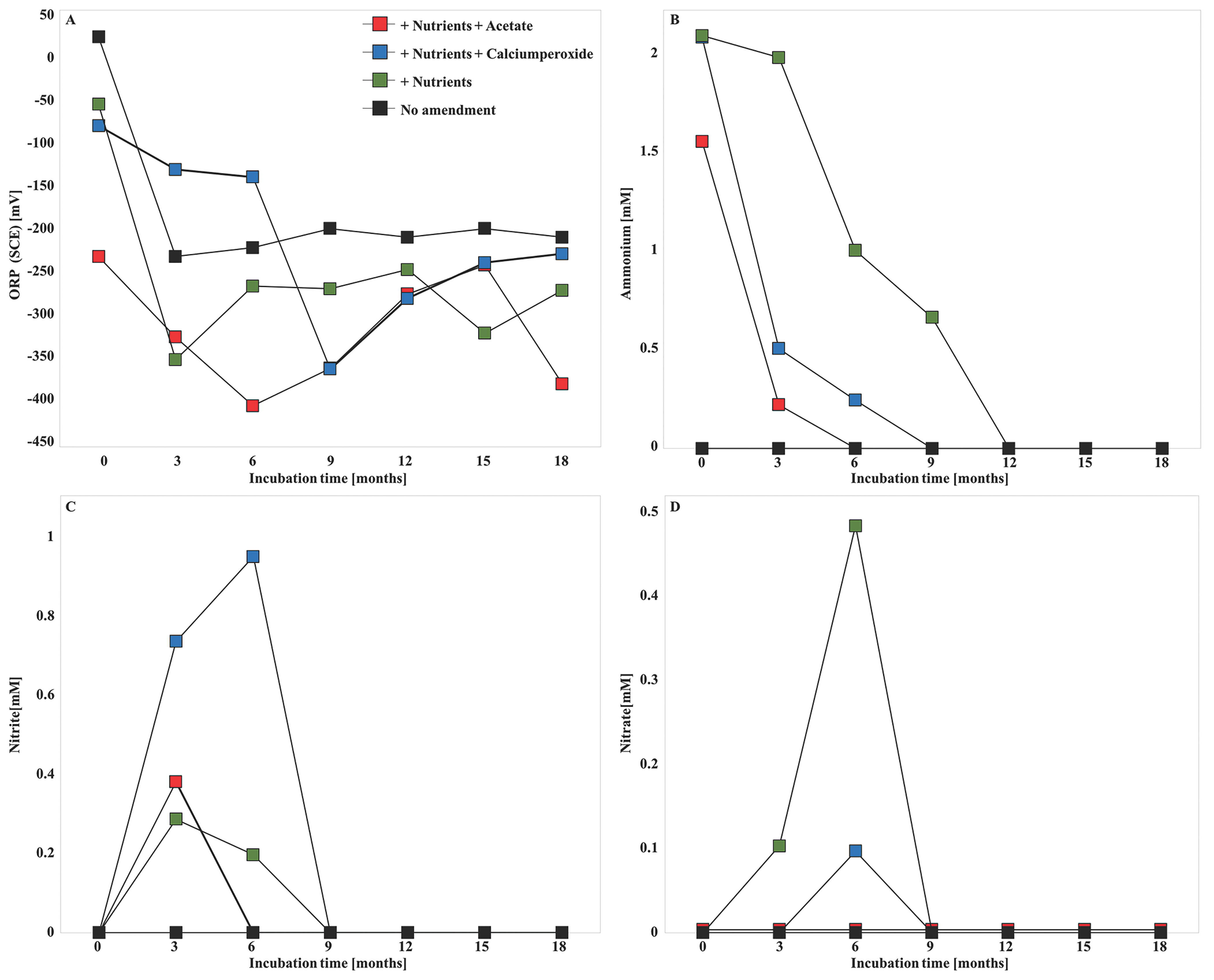
**

**Supplementary Figure 2.**

**
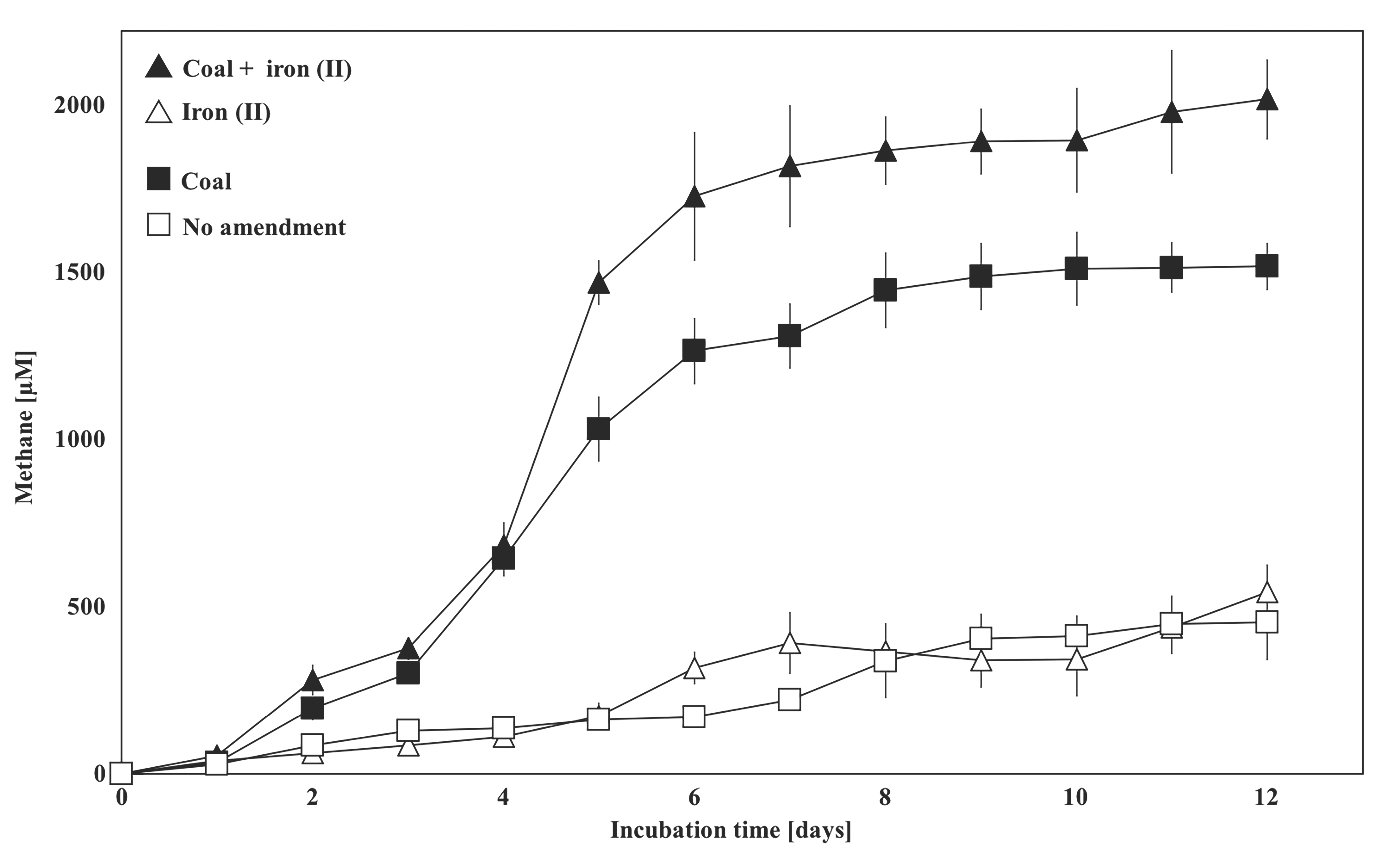
**
